# Cardiovascular‐renal axis disorder and acute‐phase proteins in cats with congestive heart failure caused by primary cardiomyopathy

**DOI:** 10.1111/jvim.15757

**Published:** 2020-05-12

**Authors:** Mengmeng Liu, Liza S. Köster, Geoffrey T. Fosgate, Christopher C. Chadwick, Íñigo Sanz‐González, Peter David Eckersall, Paul R. Wotton, Anne T. French

**Affiliations:** ^1^ Department of Small Animal Clinical Sciences Small Animal Hospital, School of Veterinary Medicine, College of Medical, Veterinary and Life Sciences, University of Glasgow Glasgow UK; ^2^ Department of Small Animal Clinical Sciences University of Tennessee, Knoxville Tennessee USA; ^3^ Faculty of Veterinary Science, Department of Production Animal Studies University of Pretoria Onderstepoort South Africa; ^4^ Life Diagnostics Inc. West Chester Pennsylvania USA; ^5^ Institute of Biodiversity, Animal Health and Comparative Medicine University of Glasgow Glasgow UK; ^6^ Department of Clinical Sciences Ross University School of Veterinary Medicine Basseterre St Kitts and Nevis

**Keywords:** acute‐phase protein, AGP, biomarker, cardiorenal syndrome, ceruloplasmin, feline cardiomyopathy, LRG1, NT‐proBNP, SAA, SDMA

## Abstract

**Background:**

Currently, the pathogenesis of congestive heart failure (CHF) in cats is not fully understood.

**Objective:**

To identify novel biomarkers for CHF in cats caused by primary cardiomyopathy, particularly related to cardiovascular‐renal axis disorder and systemic inflammatory response.

**Animals:**

Twenty‐five cats in CHF caused by primary cardiomyopathy, 12 cats with preclinical cardiomyopathy, and 20 healthy controls.

**Methods:**

Case control and observational case series. The following serum biomarkers were compared among the 3 cat groups: a cardiorenal profile that included N‐terminal pro‐brain natriuretic peptide (NT‐proBNP), symmetric dimethylarginine (SDMA), and creatinine and an inflammatory profile that included 7 acute‐phase proteins (APPs). Survival analyses and longitudinal studies were performed in CHF cats.

**Results:**

All cardiorenal biomarkers were positively correlated and higher in CHF cats, and high NT‐proBNP and SDMA were associated with poor clinical outcome. Cats with CHF had significantly higher leucine‐rich alpha‐2‐glycoprotein 1, serum amyloid A, and ceruloplasmin, and these APPs were positively correlated with NT‐proBNP and left atrial size. In a multivariable survival analysis, alpha‐1‐acid glycoprotein concentration (*P* = .01), body weight (*P* = .02) and left atrial‐to‐aortic root ratio (*P* = .01) were independent prognostic factors for CHF in these cats.

**Conclusions and Clinical Importance:**

In cats, CHF is an inflammatory disorder and outcome in CHF may be determined by the extent of inflammation and possibly the amount of residual renal function. These novel biomarkers have potential use for the clinical management, prognosis, and future research into CHF and cardiomyopathy in cats.

**Abbreviations**
AGP: alpha‐1‐acid glycoprotein
ANOVA: analysis of variance
APP: acute‐phase protein
ATE: arterial thromboembolism
AV: atrioventricular
BP: blood pressure
CHF: congestive heart failure
CI: confidence interval
CKD: chronic kidney disease
CRP: C‐reactive protein
cTnI: cardiac troponin I
CvRD: cardiovascular‐renal axis disorder
DCM: dilated cardiomyopathy
DSH: domestic short hair
GFR: glomerular filtration rate
HCM: hypertrophic cardiomyopathy
Hp: haptoglobin
HR: hazard ratio
ICU: intensive care unit
IQR: interquartile range
ISACHC: International Small Animal Cardiac Health Council
IVSd: interventricular septum thickness at end diastole
LA: left atrial
LA/Ao: left atrial‐to‐aortic root ratio
LRG1: leucine‐rich alpha‐2‐glycoprotein 1
LV FS: left ventricular fractional shortening
LVFWd: left ventricular free wall thickness at end diastole
NT‐proBNP: N‐terminal pro‐brain natriuretic peptide
PCT: procalcitonin
PE: point estimate
RCM: restrictive cardiomyopathy
ROC: receiver operating characteristic
SAA: serum amyloid A
SDMA: symmetric dimethylarginine
SEC: spontaneous echocardiographic contrast
SPARCL: spatial proximity analyte reagent capture luminescence
UCM: unclassified cardiomyopathy

## 1 INTRODUCTION

Biomarkers have drawn considerable attention in cardiovascular medicine in both the human medical and in veterinary fields. They may provide information on disease processes as well as assist in diagnosis and clinical management.^1^, ^2^ To date, commercially available cardiac biomarkers in veterinary medicine are limited to N‐terminal pro‐brain natriuretic peptide (NT‐proBNP) and cardiac troponin I (cTnI).^3^ Studies on cardiovascular‐renal axis disorder (CvRD) and inflammatory responses have the potential to identify novel biomarkers for cats with congestive heart failure (CHF).

In humans, renal dysfunction is an important predictor of heart failure outcome.^4^, ^5^, ^6^ In dogs and cats, azotemia is consistently seen in patients in CHF,^7^, ^8^, ^9^, ^10^, ^11^ with a higher incidence observed in more advanced disease.^9^, ^10^, ^11^ Although monitoring renal function is considered crucial in management of CHF,^12^ the impact of renal function on CHF outcome has not been well documented in cats. Conventionally, serum creatinine concentration is used for monitoring renal function in patients with cardiac disease as a marker of glomerular filtration rate (GFR), but its sensitivity and specificity are inferior to the newer marker of GFR, symmetric dimethylarginine (SDMA).^13^, ^14^, ^15^, ^16^ Unlike creatinine, SDMA is not affected by body lean mass,^17^ and it can detect renal dysfunction earlier than does serum creatinine concentration.^18^, ^19^ Apart from a recent study comparing SDMA in cats with hypertrophic cardiomyopathy (HCM) and primary renal disease,^2^ the biomarker role of SDMA in cats with heart disease has not been reported.

Inflammation plays an essential role in heart failure.^20^, ^21^ Acute‐phase proteins (APPs) are considered sensitive circulatory biomarkers for inflammatory conditions, and positive APPs increase in response to inflammation.^22^, ^23^ In cats, infectious diseases, neoplasia, and various systemic conditions have been associated with increased positive APPs.^24^, ^25^, ^26^, ^27^, ^28^ So far, only a few studies have investigated APPs in cats with cardiac disease.^26^, ^29^, ^30^ In humans and dogs, several APPs have been associated with heart failure.^31^, ^32^, ^33^, ^34^, ^35^, ^36^, ^37^, ^38^ The relationship between APPs and CHF in cats, however, is unclear.

Our objective was to identify novel biomarkers of CHF in cats to improve understanding of the pathogenesis of the syndrome of CHF and the implications of GFR and inflammatory status for clinical management. We investigated 2 panels of serum markers: a cardiorenal profile and an APP profile.

We hypothesized that the selected biomarkers would (1) be increased in cats with CHF; (2) be associated with survival in cats with CHF; and (3) correlate with previously established cardiac biomarkers and prognostic indicators in cats with cardiomyopathy.

## 2 MATERIALS AND METHODS

### 2.1 Study design, animals, and clinical records

The study contained both retrospective and prospective components: part 1 (baseline study): serum biomarker concentrations were compared in CHF, preclinical cardiomyopathy, and healthy control cats; and part 2 (longitudinal study): serial biomarker concentrations were assessed over time in cats with CHF.

From January 2014 to August 2017, cats diagnosed with cardiomyopathy were recruited from the Cardiology Service, University of Glasgow Small Animal Hospital, with informed owner consent. All clinical samples and data collection were approved by the Clinical Research Ethics Committee at the School of Veterinary Medicine, University of Glasgow. Inclusion criteria were primary cardiomyopathies, including HCM, dilated cardiomyopathy (DCM), unclassified cardiomyopathy (UCM), restrictive cardiomyopathy (RCM), and arrhythmogenic right ventricular cardiomyopathy. The echocardiographic diagnostic criteria for each phenotype were obtained from previous publications^39^, ^40^, ^41^, ^42^, ^43^, ^44^ (Table S7). Congestive heart failure was diagnosed based on clinical history, presentation and evidence from thoracic radiography, ultrasonography or both, including pulmonary edema, pleural effusion, pericardial effusion, and ascites. Exclusion criteria were (1) secondary cardiomyopathies associated with endocrinopathies (eg, acromegaly, hyperthyroidism) or systemic hypertension. Serum total T4 concentration was measured in all cats >6 years of age and in all cats with systolic blood pressure (BP) consistently ≥170 or ≥160 mm Hg with retinal changes suggestive of systemic hypertension^45^, ^46^; (2) a history of primary chronic kidney disease (CKD) of International Renal Interest Society stage II or higher at the time of admission; (3) preexisting conditions that have been reported to trigger an acute‐phase response, including any active infectious disease, recent inflammatory events (eg, surgery, trauma) or systemic diseases (eg, pancreatitis, neoplasia, anemia secondary to chronic inflammatory disease); and (4) incomplete diagnostic imaging records or unsatisfactory serum sample collection.

Commercial serum samples (Biobest, Edinburgh, UK) from 20 clinically healthy cats were used as controls. Echocardiographic examinations and clinical records were not available for these cats; their serum samples were screened for occult cardiac disease using NT‐proBNP, and all had concentrations <100 pmol/L.^47^, ^48^

Relevant clinical records of the cardiomyopathy cats were collected from the hospital archives or electronic information system (Excelicare, AxSys Technology, Paisley, UK). The following details were extracted for each cat at entry into the study: signalment, date and age at initial diagnosis, cardiomyopathy diagnosis, CHF diagnosis, and comorbidities. Relevant echocardiographic parameters were examined for diagnosing cardiomyopathies, and 5 of them, which have been reported to be associated with severity and prognosis of cardiomyopathy in cats, were recorded for analyses: left atrial (LA) diameter, LA‐to‐aortic root ratio (LA/Ao), left ventricular free wall thickness at end diastole (LVFWd), interventricular septum thickness at end diastole (IVSd), and left ventricular fractional shortening (LV FS).^46^, ^49^ The following clinical information was recorded at the time of blood sampling: sampling date, age at sampling, body weight, heart rhythm by auscultation, gallop sounds, heart murmur, pulse deficit, heart rate, respiratory rate, ECG findings, CHF severity score based on International Small Animal Cardiac Health Council (ISACHC) classification^50^, ^51^ (IIa = 1; IIb = 2; IIIa = 3; IIIb = 4), cTnI (if measured for diagnostic purpose), and cardiac medications.

Survival information of the CHF cats was collected from the hospital record system or by contacting the referring veterinary practice or client. For cats that died, survival in days was defined as the time between initial diagnosis and the date of death. For cats still alive at the end of the study or lost to follow‐up, date of last contact was used as the censoring time in the statistical analysis. Causes of mortality were recorded and divided into cardiac and noncardiac causes. The cardiac causes included cats that died or were euthanized because of cardiac disease. Overall CHF stability during the study period was summarized for each cat. A “stable” status was defined as 1 of 2 scenarios: a cat in ISACHC class IIIa that was stable on treatment and did not redevelop heart failure during the study, or a cat that was in ISACHC class IIIb heart failure at the time of inclusion into the study that did not require rehospitalization. A “nonstable” status was defined as a cat that reached a cardiac event. An event was defined as either death (cardiac death or euthanasia prompted by cardiac disease that substantially affected quality of life), progression in ISACHC class, or when hospitalization was required at each visit. Other recorded information included frequency of thoracocentesis, total days in the intensive care unit (ICU), spontaneous echocardiographic contrast (SEC), an intracardiac thrombus seen on echocardiography, arterial thromboembolism (ATE), and cardiac medications received during the study.

### 2.2 Blood sampling, preparation, and storage

Blood samples were collected from cardiomyopathy cats as soon as possible after initial diagnosis. For the CHF cats enrolled in the longitudinal study, subsequent sample collections were performed at follow‐up revisits. Blood was sampled by venipuncture for clinical diagnostic purpose with an additional volume of 0.3‐1.5 mL blood obtained for serum biomarker measurements. Following collection, blood samples were allowed to clot and then centrifuged at 9000 rpm for 3 minutes at room temperature. Serum was separated and transferred to a new collection tube and stored at −20°C before transfer to a −80°C freezer for later analysis. Control serum samples from healthy cats were stored using the same protocol.

### 2.3 Measurement of CvRD biomarkers and APPs

Ten serum markers were examined, including 3 biomarkers associated with CvRD (cardiac marker NT‐proBNP, renal markers SDMA, and creatinine) and 7 APPs: alpha‐1‐acid glycoprotein (AGP), C‐reactive protein (CRP), haptoglobin (Hp), leucine‐rich alpha‐2‐glycoprotein 1 (LRG1), serum amyloid A (SAA), procalcitonin (PCT), and ceruloplasmin.

For CvRD marker testing, 200‐330 μL aliquots of serum were shipped to the IDEXX Reference Laboratory (Ludwigsburg, Germany). The NT‐proBNP concentration was measured using the Cardiopet proBNP assay, SDMA was measured using IDEXX SDMA (enzyme immunoassay), and creatinine was measured using a kinetic color test (compensated Jaffé reaction). In addition, NT‐proBNP concentrations from 9 cats (3 CHF and 6 preclinical cardiomyopathy), sampled and measured in 2014, were included retrospectively. These measurements used the same methodology and the test was performed in the IDEXX Reference Laboratory.

The APPs were measured by Life Diagnostic Inc. (Goshen, Pennsylvania); serum samples were shipped on dry ice. Seven spatial proximity analyte reagent capture luminescence (SPARCL) assays (Life Diagnostic Inc.) were used for APP measurements (Table 1). Before sample testing, lyophilized APP stock was reconstituted with diluent (CSD50‐1, Life diagnostic Inc.), and 8 standards were prepared for each APP. Depending on the assay, serial dilutions were performed following datasheet recommendations. Serum samples were diluted to different concentrations (Table 1) depending on the APP. In a 96‐well plate, 25 μL of mixed horseradish peroxidase and acridan‐conjugated antibody were added, followed by 50 μL standard solution or diluted sample. The plate was incubated for 30 minutes at 25°C and 150 rpm on a microplate incubator/shaker. After incubation, the plate was placed in a preset plate reader BMG LUMI star Omega luminometer (BMG LABTECH, Cary, North Carolina); sample luminescence was measured immediately after adding 37.5 μL of trigger solution. Standard curves were calculated by plotting standard luminescence versus log 10 of the standard concentration. Sample APP concentration was calculated from the standard curve by converting the luminescence data antilog.

**TABLE 1:** SPARCL assays used in the APP measurements

APP	Catalogue number	Species	Sample dilution (fold)
AGP	AGP‐SP‐8	Feline	20 000
CRP	CRP‐SP‐8	Feline	125 000
Hp	Hapt‐SP‐8	Feline	50 000
SAA	SAA‐SP‐8	Feline	1000 or 50 000
PCT	PCT‐SP‐4	Canine	40
LRG1	LRG‐SP‐8	Feline	100
Ceruloplasmin	CER‐SP‐8	Feline	10 000

Abbreviations: AGP, alpha‐1‐acid glycoprotein; APP, acute‐phase protein; CRP, C‐reactive protein; Hp, haptoglobin; LRG1, leucine‐rich alpha‐2‐glycoprotein 1; PCT, procalcitonin; SAA, serum amyloid A; SPARCL, spatial proximity analyte reagent capture luminescence.

### 2.4 Data analyses

Before statistical analysis, data were assessed for normality using an Anderson‐Darling test, and natural logarithm, square root, or rank transformations subsequently were used to normalize raw data distributions. Clinical variables of CHF and preclinical cats at admission were analyzed using 1‐way analysis of variance (ANOVA) or *t* tests for quantitative data comparison and chi‐square or Fisher exact tests for categorical data. Baseline biomarker concentrations were analyzed using ANOVA followed by multiple pairwise *t* tests, with Bonferroni correction of post hoc *P* values. The correlation between biomarkers and clinical variables was assessed using scatter plots and calculating Spearman's rho.

Candidate prognostic markers were compared between surviving and dead CHF cats using independent t tests. Cox proportional hazards' models were used to estimate the association between biomarkers and other variables with survival time in CHF cats. Univariate screening models were used and variables with significance at *P* < .20 were selected for multivariable modeling employing a backwards stepwise process. Spearman's rho was used to evaluate collinearity between selected variables. When variables were collinear, the variable with the weaker univariate association with survival was excluded from the multivariable model. Significant variables were analyzed using receiver operating characteristic (ROC) curve analysis to determine the most accurate cutoff for identifying the nonsurviving cats based on the largest Youden index. Variables were removed from multivariable models one‐by‐one based on the largest Wald *P* value until all remaining variables were significant at *P* < .05. Interaction terms were not evaluated.

The following commercially available software was used for statistical analysis: MINITAB Statistical Software Release 13.32 (Minitab Inc., State College, Pennsylvania), Epi Info Version 6.04 (CDC, Atlanta, Georgia), IBM SPSS Statistics Version 24 (International Business Machines Corp., Armonk, New York), and Microsoft Office Excel (Microsoft Corporation, Redmond, Washington).

Statistical significance was set at *P* < .05.

## 3 RESULTS

### 3.1 Study population

In total, 37 cardiomyopathy cats were enrolled in the study after exclusion, with 25 in CHF and 12 in preclinical stage. Signalment, body weight, and clinical information are listed in Table 2. Healthy control cats were significantly younger than the CHF and preclinical cardiomyopathy cats (*P* = .003). Domestic short hair and male cats were overrepresented for cardiomyopathy. No significant differences in sex, age and body weight were found between CHF and preclinical cats. Hypertrophic cardiomyopathy was the most common phenotype in both CHF and preclinical groups. An obstructive form of HCM^46^ was diagnosed in 4 CHF and 8 preclinical cats. Compared with the preclinical group, cats in the CHF group had significantly higher respiratory rates, higher LA diameter, higher LA/Ao ratio, and lower LV FS% (Table 3).

**TABLE 2:** Signalment, body weight, and diagnoses in the study populations

	CHF (n = 25)	Preclinical (n = 12)	Healthy (n = 20)
Age (y)	8.1 ± 4.7 (1.0‐15.3)	6.6 ± 4.4 (0.7‐14.3)	4.3 ± 3.0 (0.6‐11.3)
Breed	DSH (n = 21); British blue, Ragdoll, Siamese, Bengal (n = 1 each)	DSH (n = 12)	NA
Sex	Female (n = 8); male (n = 17)	Female (n = 3); male (n = 9)	Female (n = 10); male (n = 10)
Weight (kg)	4.9 ± 1.7 (2.3‐9.5)	5.7 ± 1.4 (3.7‐8.0)	NA
Cardiomyopathy diagnosis	44% HCM; 28% UCM; 24% RCM; 4% DCM	92% HCM; 8% UCM	NA
CHF diagnosis	Pulmonary edema (n = 13); pleural effusion (n = 17); pericardial effusion (n = 6); ascites (n = 4)	NA	NA
Comorbidities	Diabetes mellitus, tricuspid dysplasia, complete AV bock with pacemaker implanted (n = 1 each); respiratory disease (n = 2)	Chronic benign mass, eosinophilia of unknown origin (n = 1 each); mild periodontal disease (n = 2)	NA

*Note:* Age and body weight are presented as mean ± SD (range). In cardiomyopathy cats, 32 cats were neutered.

Abbreviations: AV, atrioventricular; CHF, congestive heart failure; DCM, dilated cardiomyopathy; DSH, domestic short hair; HCM, hypertrophic cardiomyopathy; F, female; M, male; NA, not available/applicable; RCM, restrictive cardiomyopathy; UCM, unclassified cardiomyopathy.

**TABLE 3:** Comparison of clinical variables in cardiomyopathy cats at admission

Variable	CHF (n = 25)		Preclinical (n = 12)		*P* value
	n/d	PE^a^ (Interval^b^)	n/d	PE^a^ (Interval^b^)	
Irregular heart rhythm	10/18	0.56 (0.33, 0.77)	1/7	0.14 (0.01, 0.53)	.09
Gallop sounds audible	4/24	0.17 (0.06, 0.35)	0/12	0.00 (0.00, 0.22)	.28
Murmur present	12/19	0.63 (0.40, 0.82)	11/12	0.92 (0.65, 1.0)	.11
Murmur grade ≥3	9/19	0.47 (0.26, 0.69)	6/12	0.50 (0.23, 0.77)	.89
Pulse deficit	3/11	0.27 (0.07, 0.58)	1/8	0.13 (0.01, 0.48)	.60
Abnormal ECG	13/20	0.65 (0.43, 0.83)	3/9	0.33 (0.09, 0.67)	.23
Heart rate (per min)	24/25	180 (165, 200)	12/12	169 (160, 195)	.40
Respiratory rate (per min)	25/25	44 (36, 60)	12/12	30 (20, 55)	.01**
LA diameter (mm)	25/25	20.0 (16.5, 22.0)	12/12	13.0 (11.3, 15.0)	<.001***
LA/Ao ratio	25/25	2.34 (1.84, 2.56)	12/12	1.32 (1.26, 1.39)	<.001***
LVFWd (mm)	25/25	6.38 (5.21, 7.41)	12/12	6.15 (5.03, 7.67)	.99
IVSd (mm)	25/25	6.03 (4.54, 7.53)	12/12	5.23 (4.67, 7.73)	.46
LV FS (%)	25/25	37.0 (23.5, 47.0)	12/12	53.0 (44.5, 63.0)	.001**

Abbreviations: CHF, congestive heart failure; IVSd, interventricular septum thickness at end diastole; LA, left atrial; LA/Ao ratio, left atrial‐to‐aortic root ratio; LV FS, left ventricular fractional shortening; LVFWd, left ventricular free wall thickness at end diastole; n/d, numerator/denominator; PE, point estimate.

a PE is corresponding to the proportion for categorical variables and the median for quantitative data.

b Interval: 95% confidence interval for categorical data and interquartile range for quantitative data.

** Statistical significance at *P* < .01; ***statistical significance at *P* < .001.

At admission, all CHF cats were in ISACHC class IIIa or IIIb (ratio 2 : 1). The CHF presentations are listed in Table 2. The CHF cats received combinations of cardiac medications at enrollment, including furosemide (1.0‐13.8 mg/kg/24 h, IV or PO), torsemide (0.2‐1.0 mg/kg/24 h, PO), benazepril (0.2‐1.0 mg/kg/24 h, PO), spironolactone (1.3‐2.6 mg/kg/24 h, PO), pimobendan (0.3‐0.7 mg/kg/24 h, IV or PO), clopidogrel (1.9‐8.9 mg/kg/24 h, PO), aspirin (3.6‐8.0 mg/kg/72 h, PO), diltiazem (5.2‐7.7 mg/kg/24 h, PO), sotalol (4.3‐4.8 mg/kg/24 h, PO), and potassium supplementation. Arrhythmias included atrial fibrillation (n = 4), ventricular tachycardia (n = 1), atrial and ventricular premature complexes (n = 12), and complete atrioventricular (AV) block (n = 1). Nine cats had SEC, 1 had an intracardiac thrombus, and 1 had a history of suspected ATE (resolved before admission). During the study period, thoracocentesis was performed in 10 cats (1‐4 times), 17 cats required stabilization in the ICU with a total hospitalization time of 4.3 ± 3.6 days (range, 0.5‐15 days), and 11 cats overall were stable for CHF with the other 14 cats being classified as unstable. By November 2017, 12 cats in CHF were alive and 13 cats had died, with median survival time from initial diagnosis of 132 days (interquartile range [IQR], 48‐189) and 29 days (IQR, 13‐119), respectively. Cardiac death was confirmed in 8 cats.

### 3.2 Baseline biomarker concentrations in CHF, preclinical cardiomyopathy, and healthy control cats

**FIGURE 1:**
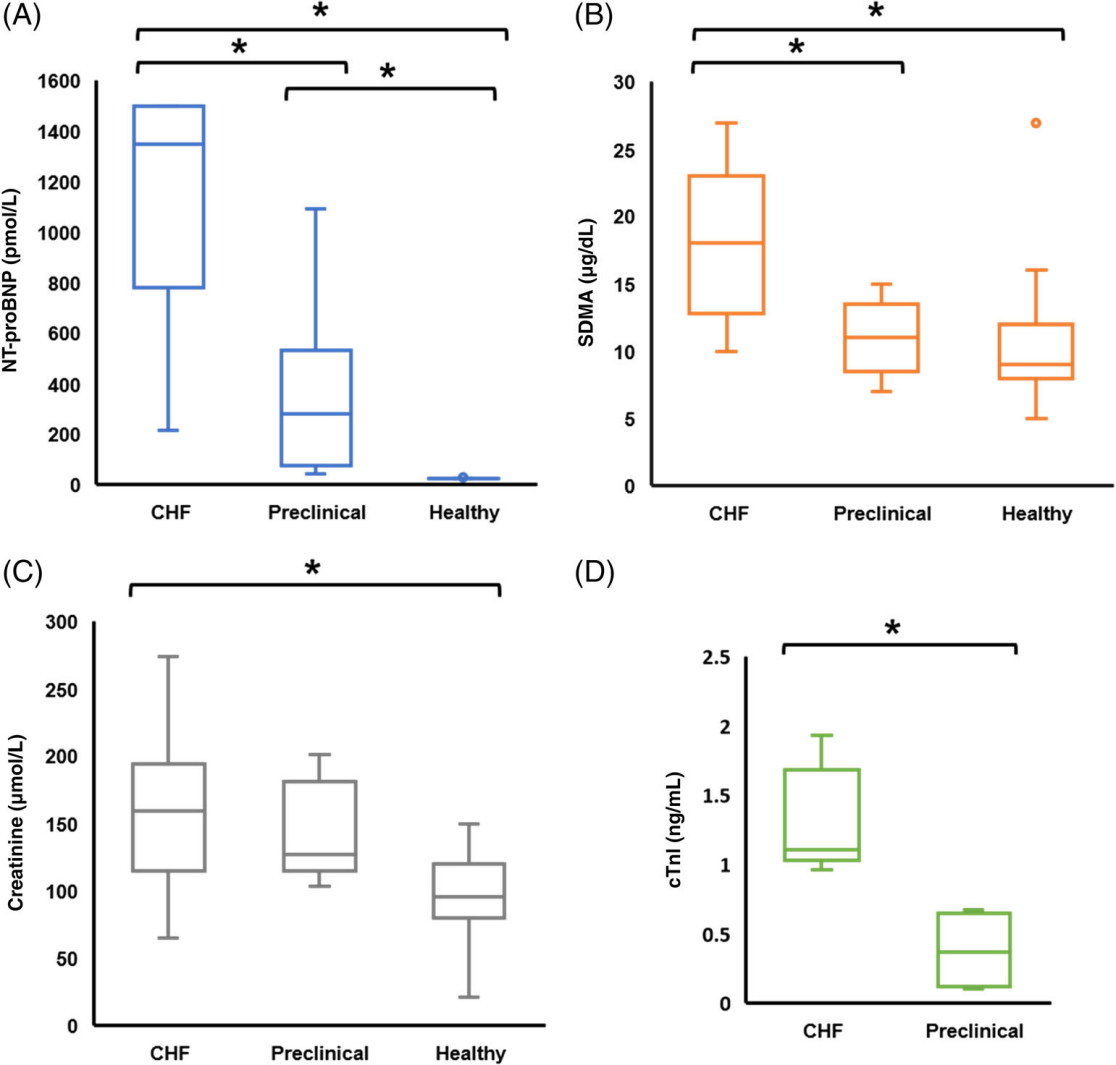
CHF cats had significantly higher circulating CvRD biomarker levels than controls. NT‐proBNP clearly differentiated CHF (n = 23), preclinical (n = 11), and healthy controls (n = 20); SDMA and creatinine were increased in CHF cats (n = 18) compared with preclinical (n = 5) and healthy controls (n = 19). Clinically measured cTnI was higher in CHF cats (n = 5) compared to preclinical cats (n = 4). Boxes represent data IQR between 25% and 75%, with a horizontal bar in each box representing median value; the space between T‐bars extended from the box indicates the full data range; circle indicates an outlier. Asterisk (*) indicates statistical significance at *P* < .05. CHF, congestive heart failure; cTnI, cardiac troponin I; CvRD, cardiovascular‐renal axis disorder; IQR, interquartile range; NT‐proBNP, N‐terminal pro‐brain natriuretic peptide; SDMA, symmetric dimethylarginine

All CvRD biomarker concentrations were higher in CHF cats compared to healthy controls. The NT‐proBNP and SDMA concentrations were higher in cats with CHF compared to those with preclinical cardiomyopathy (Figure 1). The NT‐proBNP concentration also significantly differentiated preclinical cats from healthy controls. In the CHF group, 61% of cats had abnormal serum SDMA concentration (>14 μg/dL), and 44% had abnormal serum creatinine concentration (IDEXX laboratory reference range, <168 μmoL/L).

**FIGURE 2:**
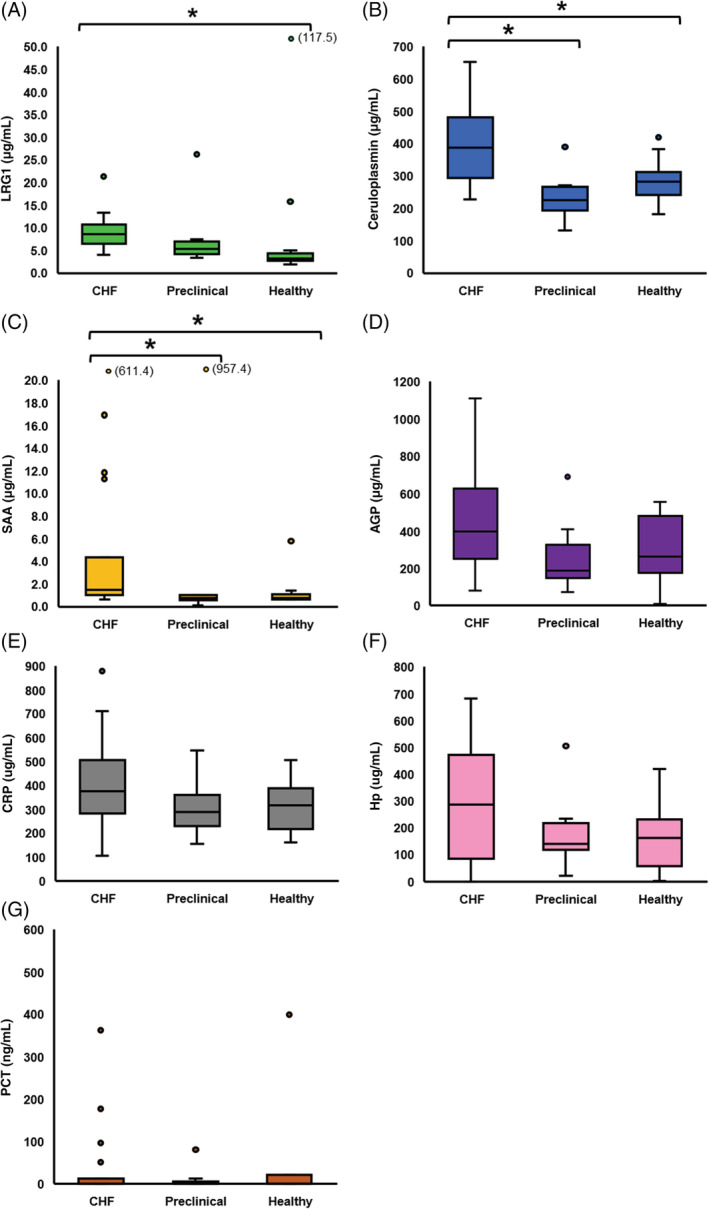
CHF cats (n = 15) had significantly higher LRG1, SAA, and ceruloplasmin than healthy controls (n = 16); significantly higher SAA and ceruloplasmin than preclinical cats (n = 9). No significant differences in AGP, CRP, Hp, and PCT among the three cat groups (*P* > .05). Boxes represent data IQR between 25% and 75% with a horizontal bar in each box representing median value; the space between T‐bars extended from the box indicates full data range; circles indicate outliers, 3 outliers exceeding the vertical scale range are labeled with original values. Asterisk (*) indicates statistical significance at *P* < .05. AGP, alpha‐1‐acid glycoprotein; CHF, congestive heart failure; CRP, C‐reactive protein; Hp, haptoglobin; IQR, interquartile range; LRG1, leucine‐rich alpha‐2‐glycoprotein 1; PCT, procalcitonin; SAA, serum amyloid A

Concentrations of 3 APPs (LRG1, SAA, and ceruloplasmin) were significantly higher in CHF cats compared to healthy controls (Figure 2A‐C). Among these, SAA and ceruloplasmin were higher in CHF cats compared to cats with preclinical cardiomyopathies. No significant differences in AGP, CRP, Hp, and PCT were found among the 3 cat groups (Figure 2D‐G). The concentration of PCT was zero in most cats (24 of 37), regardless of cardiac disease status.

### 3.3 Correlations between biomarkers and clinical variables

The cardiorenal biomarkers were positively correlated with each other (*P* < .05; Table 4). The correlations among NT‐proBNP, SDMA, and creatinine were moderate (*r* = 0.401‐0.615), and correlation between NT‐proBNP and cTnI was strong (*r* = 0.73).

**TABLE 4:** Correlation between measured biomarkers and clinical variables at each sampling period in all cats participated in the study

	NT‐proBNP	SDMA	Creatinine	AGP	CRP	Hp	LRG1	SAA	PCT	Ceruloplasmin
NT‐proBNP	1	**0**.**615** (<.001)	**0.454** (<.001)	**0.269** (.05)	0.240 (.08)	0.242 (.08)	**0.456** (.001)	**0.317** (.02)	−0.168 (.22)	**0.439** (.001)
SDMA	**0.615** (<.001)	1	**0.401** (.002)	0.277 (.06)	**0.543** (<.001)	**0.417** (.004)	0.210 (.18)	0.254 (.09)	**−0.385** (.01)	0.174 (.25)
Creatinine	**0.454** (<.001)	**0.401** (.002)	1	0.116 (.45)	**0.312** (.04)	0.174 (.25)	**0.470** (.001)	**0.472** (.001)	−0.043 (.78)	0.232 (.12)
AGP	**0.269** (.05)	0.277 (.06)	0.116 (.45)	1	0.145 (.29)	**0.421** (.001)	**0.288** (.03)	**0.474** (<.001)	−0.053 (.70)	**0.610** (<.001)
CRP	0.240 (.08)	**0.543** (<.001)	**0.312** (.04)	0.145 (.29)	1	0.257 (.06)	**0.394** (.003)	**0.402** (.002)	−0.134 (.33)	**0.291** (.03)
Hp	0.242 (.08)	**0.417** (.004)	0.174 (.25)	**0.421** (.001)	0.257 (.06)	1	0.193 (.16)	**0.314** (.02)	**−0.325** (.01)	**0.327** (.02)
LRG1	**0.456** (.001)	0.210 (.17)	**0.470** (.001)	**0.288** (.03)	**0.394** (.003)	0.193 (.16)	1	**0.481** (<.001)	**0.420** (.001)	**0.342** (.011)
SAA	**0.317** (.02)	0.254 (.09)	**0.472** (.001)	**0.474** (<.001)	**0.402** (.002)	**0.314** (.02)	**0.481** (<.001)	1	−0.022 (.87)	**0.527** (<.001)
PCT	−0.168 (.22)	**−0.385** (.01)	−0.043 (.78)	−0.053 (.70)	−0.134 (.33)	**−0.325** (.02)	**0.420** (.001)	−0.022 (.87)	1	0.085 (.54)
Ceruloplasmin	**0.439** (.001)	0.174 (.25)	0.232 (.12)	**0.610** (<.001)	**0.291** (.03)	**0.327** (.08)	**0.342** (.011)	**0.527** (<.001)	0.085 (.54)	1
cTnI	**0.730** (.08)	0.667 (.22)	0.700 (.19)	**0.786** (.04)	−0.071 (.88)	**0.893** (.01)	0.750 (.05)	0.559 (.19)	0.236 (.61)	0.643 (.12)
LA diameter	**0.528** (.001)	0.324 (.13)	0.165 (.45)	0.300 (.12)	0.297 (.12)	**0.422** (.02)	**0.537** (.003)	**0.420** (.03)	0.053 (.79)	**0.429** (.02)
LA/Ao ratio	**0.547** (.001)	0.404 (.06)	0.110 (.62)	0.150 (.45)	0.313 (.10)	0.309 (.11)	**0.445** (.02)	0.147 (.46)	0.146 (.46)	**0.376** (.05)
LVFWd	0.133 (.45)	0.030 (.89)	**−0.453** (.03)	0.174 (.38)	0.099 (.62)	0.208 (.29)	−0.109 (.58)	0.243 (.21)	**−0.562** (.002)	0.204 (.30)
IVSd	0.084 (.64)	0.072 (.74)	−0.004 (.99)	0.224 (.25)	0.239 (.22)	−0.007 (.97)	−0.011 (.96)	0.204 (.30)	−0.216 (.27)	0.193 (.33)
LV FS	**−0.428** (.01)	−0.362 (.09)	**−0.468** (.02)	−0.171 (.38)	−0.333 (.08)	−0.302 (.19)	**−0.388** (.04)	−0.229 (.24)	0.003 (.99)	−0.201 (.31)
CHF grade	**0.489** (<.001)	0.305 (.05)	−0.035 (.83)	0.309 (.06)	0.044 (.80)	0.221 (.18)	0.213 (.20)	**0.345** (.03)	0.079 (.64)	**0.405** (.01)
Age (sampling)	0.183 (.12)	0.178 (.17)	**0.505** (<.001)	0.059 (.67)	0.175 (.20)	0.162 (.24)	**0.296** (.03)	**0.363** (.01)	0.136 (.32)	0.247 (.07)

*Note:* Data presented as Spearman's rank correlation (*P* value). Significant correlations are presented in bold font.

Abbreviations: AGP, alpha‐1‐acid glycoprotein; CRP, C‐reactive protein; cTnI, cardiac troponin I; Hp, haptoglobin; ISACHC, International Small Animal Cardiac Health Council; LA, left atrial; LA/Ao, left atrial‐to‐aortic root ratio; LRG1, leucine‐rich alpha‐2glycoprotein1; NT‐proBNP, N‐terminal pro‐brain natriuretic peptide; PCT, procalcitonin; SAA, serum amyloid A; SDMA, symmetric dimethylarginine.

The APPs LRG1, SAA, ceruloplasmin, and AGP were positively correlated with the 2 cardiac biomarkers, LA size, and CHF class (*P* < .05; Table 4). Alpha‐1‐acid glycoprotein was strongly correlated with cTnI (*r* = 0.786); LRG1, SAA, and ceruloplasmin had moderate correlation with LA/Ao ratio, LA diameter or both (*r* = 0.376‐0.537); SAA and ceruloplasmin were moderately correlated with CHF severity (*r* = 0.345 and 0.405, respectively).

### 3.4 Survival analysis in CHF cats

For all‐cause mortality, 5 variables determined at admission were significantly different in cats that died compared with cats that survived (Table 5). These included increased LA diameter and LA/Ao ratio, decreased LV FS, increased NT‐proBNP, and increased SDMA.

**TABLE 5:** Comparison of potential prognostic indicators for CHF cats

Variable	Survived (n = 12)		Died (n = 13)		*P* value
	Median (n)	IQR	Median (n)	IQR	
Age at diagnosis (y)	9.6 (12)	4.9, 12.2	5.1 (13)	4.1, 13.1	.91
Weight (kg)	4.9 (11)	4.3, 6.8	3.8 (13)	3.3, 5.4	.05
LA diameter (mm)	18.0 (12)	16.3, 21.0	21.0 (13)	17.5, 22.5	.003**
LA/Ao ratio	2.11 (12)	1.72, 2.54	2.36 (13)	2.03, 2.86	<.001***
LVFWd (mm)	6.15 (12)	5.44, 7.37	6.56 (13)	4.60, 7.70	.73
IVSd (mm)	6.37 (12)	4.37, 8.94	6.00 (13)	4.68, 7.21	.89
LV FS (%)	37.5 (12)	26.5, 48.3	30.0 (13)	21.0, 46.5	.04*
NT‐proBNP (pmol/L)	780 (11)	578, 1477	>1500 (12)	1308, >1500	.001**
SDMA (μg/dL)	13.0 (9)	11.0, 20.0	21.0 (9)	16.5, 23.0	.01**
Creatinine (μmol/L)	157 (9)	114, 201	161 (9)	111, 191	.92
AGP (μg/mL)	302 (9)	234, 477	459 (10)	248, 770	.09
CRP (μg/mL)	476 (9)	274, 544	368 (10)	267, 439	.94
Hp (μg/mL)	272 (9)	145, 361	307 (10)	24, 641	.53
LRG1 (μg/mL)	9.4 (9)	7.8, 11.9	8.6 (10)	5.5, 11.3	.94
SAA (μg/mL)	1.70 (9)	1.23, 14.09	1.11 (10)	0.84, 3.45	.84
PCT (ng/mL)	0 (9)	0, 54.0	0 (10)	0, 21.9	.94
Ceruloplasmin (μg/mL)	391 (9)	283, 487	372 (10)	289, 496	.93

*Note:* Age, weight, five echocardiographic variables and ten serum biomarkers were compared between survived and dead CHF cats.

Abbreviations: AGP, alpha‐1‐acid glycoprotein; CHF, congestive heart failure; CRP, C‐reactive protein; Hp, haptoglobin; IQR, interquartile range; IVSd, interventricular septum thickness end diastole; LA, left atrial; LA/Ao ratio, left atrial‐to‐aortic root ratio; LRG1, leucine‐rich alpha‐2‐glycoprotein 1; LV FS, left ventricular fractional shortening; LVFWd, left ventricular free wall thickness end diastole; NT‐proBNP, N‐terminal pro‐brain natriuretic peptide; PCT, procalcitonin; SAA, serum amyloid A; SDMA, symmetric dimethylarginine.

* Statistical significance at *P* < .05; **Statistical significance at *P* < .01; ***Statistical significance at *P* < .001.

In univariate Cox proportional hazard models, significant risk predictors included unstable CHF (*P* = .02), an arrhythmia detected by auscultation (*P* = .02), gallop sounds (*P* = .03), increased LA/Ao ratio (*P* = .007), and increased serum AGP concentration (*P* = .007; Table S3). Final multivariable survival analysis (Table 6) identified body weight (*P* = .02), LA/Ao ratio (*P* = .01), and serum AGP concentration (*P* = .009) as independent poor prognostic factors in CHF cats.

**TABLE 6:** Multivariable Cox proportional hazards analysis evaluating the effects of potential prognostic factors for CHF cats

Variable/level	N	PE ($\hat{\beta }$)	HR (95% CI)	*P* value (Wald)
Age				
<7 y	10	1.482	4.40 (0.39, 49.5)	.23
≥7 y	9	Referent		
Sex				
Male	13	−0.528	0.59 (0.07, 5.19)	.63
Female	6	Referent		
Weight				
<4.5 kg	10	3.891	49.0 (1.71, 1402)	.02*
≥4.5 kg	9	Referent		
LA/Ao ratio				
≥2	12	4.682	108 (2.66, 4395)	.01*
<2	7	Referent		
AGP				
≥600 (μg/mL)	5	3.695	40.2 (2.53, 641)	.01**
<600 (μg/mL)	14	Referent		

*Note:* Six cats had missing data in one or more of the analyzed variables and therefore could not be included.

Abbreviations: AGP, alpha‐1‐acid glycoprotein; CHF, congestive heart failure; CI, confidence interval; HR, hazard ratio; LA/Ao ratio, left atrial‐to‐aortic root ratio; N, number; PE, parameter estimate.

* Statistical significance at *P* < .05; **statistical significance at *P* < .01.

### 3.5 Longitudinal biomarker study in CHF cats

From August 2016 to August 2017, 12 CHF cats had 2‐5 serial blood samples collected over a time interval from 7 to 321 days after the first sample collection date. By November 2017, 6 of these cats had died. Descriptive preliminary data are summarized as follows: (1) 67% of these cats had an initial NT‐proBNP concentration >1500 pmol/L. This finding occurred in 100% of the cats that died, with 67% of them having persistent NT‐proBNP concentration >1500 pmol/L. In the survivor group, 80% of cats had stable (**<**60% increase) or decreased (**>**60% decrease) NT‐proBNP concentrations^52^ in follow‐up blood samples, (2) temporal changes in serum SDMA and creatinine concentrations were predominantly consistent with each other, but 8 conflicting data pairs occurred with abnormal serum SDMA but normal serum creatinine concentrations. One cat developed an abnormal serum creatinine concentration in addition to abnormal SDMA concentration after approximately 6 months. Cats with a single SDMA concentration >20 μg/dL all died before the end of the study, and (3) longitudinal changes in APPs were highly diverse among individual cats, with no clear patterns appreciated, although most APPs decreased after the initial measurements. Survivors and nonsurvivors had no apparent differences in serial APP measurements.

## 4 DISCUSSION

The results of our study indicate that both CvRD and an inflammatory response occurred in cats with CHF caused by primary cardiomyopathy. Secondly, several biomarkers were associated with a poor outcome and have the potential to be considered as survival prognosticators in cats with CHF, warranting further investigation.

The presence of CvRD in CHF cats was supported by simultaneous increases and correlations of cardiac biomarker NT‐proBNP and renal biomarkers, SDMA, and creatinine. This finding suggests a close functional relationship between the heart and kidney in heart failure, which has been described in other species.^5^, ^6^, ^53^ Surrogate markers of glomerular filtration (creatinine and SDMA) were increased in approximately 50% of the cats with CHF in our study, consistent with previous reports in which the prevalence of azotemia in cats with CHF was estimated to be 53%‐59%.^7^, ^8^ The high prevalence of azotemia in CHF is thought to be a reflection of CvRD, which can result either from intrinsic renal insufficiency or the iatrogenic consequences of medical management.^54^ In our study we could not differentiate the 2 causes, because all CHF cats were treated at enrollment. The use of diuretics and angiotensin‐converting enzyme inhibitors can cause prerenal azotemia as well as renal injury.^55^ It is possible that the increased serum SDMA and creatinine concentrations in CHF cats were mainly caused by higher diuretic doses in more severe CHF cases, but we did not detect any significant correlations between diuretic dose and renal markers in 13 CHF cats in a preliminary study (data not shown). Cardiovascular‐renal disorder might not be present in the early stages of cardiomyopathy in cats, at least based on currently used marker panels, because we did not identify significant differences in serum SDMA and creatinine concentrations between preclinical cardiomyopathy cats and healthy controls. Similarly, a previous publication showed no significant increase in serum SDMA concentration in predominantly preclinical HCM cats.^2^ Renal injury markers with higher sensitivity for detecting CvRD would be useful to investigate in the preclinical population.^54^

Both baseline and longitudinal study results in CHF cats supported SDMA being more sensitive than creatinine, a finding that is consistent with previous publications.^18^, ^19^, ^56^ Compared with creatinine, even with less specificity for CKD,^18^ SDMA has 2 advantages: (1) it is a more accurate renal function marker in cachexic cardiac patients with low lean body mass and (2) its superior sensitivity would allow earlier detection of changes in kidney function in acute heart failure, when secondary acute renal injury may still be reversible if prompt action is taken.^54^ Our findings support the measurement of serum SDMA concentration in cats with CHF for the purpose of early detection of CvRD and appropriate intervention.

Symmetric dimethylarginine also may have prognostic value in cats with CHF, because in our study abnormal serum SDMA concentrations appeared to be associated with shorter survival. Interestingly, multiple studies in humans have demonstrated that SDMA has value as an independent risk predictor for all‐cause mortality and cardiovascular disease.^57^ Also, SDMA might play an active role in endothelial dysfunction,^58^ which also can contribute to cardiorenal disorders.^59^, ^60^ Endothelial function was not evaluated in our study and further research is warranted.

Circulating APPs are highly sensitive inflammatory biomarkers but lack specificity, and a multiple APP‐based profile has been recommended for optimal assessment of inflammatory conditions.^61^ Our study had a relatively small sample size, but using a multiple APP screening approach, we identified 4 of 7 APPs to be associated with CHF in cats.

As a novel APP, the biological functions of LRG1 are not clear. It has been reported to be associated with inflammation and might play a role in myocardial fibrosis by interacting with transforming growth factor beta.^35^ In mice, LRG1 has a protective function against adverse cardiac remodeling in experimentally induced myocardial infarction.^53^ In our study, LRG1 was significantly increased in CHF cats. The positive correlations with both LA size and NT‐proBNP suggest that LRG1 may be associated with cardiac remodeling in cats with CHF. Myocardial and extracellular matrix remodeling are common histopathological findings in cats with cardiomyopathy.^40^, ^62^, ^63^ Our data indicate a need to determine if serum LRG1 concentration reflects changes in cardiac expression or clearance in cats with CHF, and if a direct role in cats can be identified. Investigations of LRG1 and its related signaling pathways may offer novel therapeutic targets in cats with cardiomyopathy, and LRG1 also may have a role as an additional cardiac biomarker in cats.

Serum amyloid A protein is a major APP that previously was reported to be increased in sick, hospitalized cats,^25^, ^64^ and was an independent prognostic factor in sick cats, regardless of cause.^26^ In our study, increases in SAA concentration were associated with advanced CHF but not preclinical cardiomyopathy. A recent report found significant increases of SAA concentration in preclinical HCM cats, particularly in those with generalized left ventricular hypertrophy compared to those with focal hypertrophy, suggesting a possible association of SAA in the early stages of certain types of HCM.^30^ In our study, such an association was not found, but it should be noted that our preclinical cardiomyopathies were not restricted to HCM phenotype and more than one third of them did not have generalized LV hypertrophy, which may explain the discrepancy between the 2 studies.

Ceruloplasmin is a copper transporter involved in iron detoxification and oxidative stress,^22^, ^65^ and it was significantly higher in the CHF cats. Both SAA and ceruloplasmin were positively correlated with NT‐proBNP, LA size, and CHF grade, which suggests these 2 APPs potentially could be used for assessing disease progression and staging cats with CHF.

To date, AGP has not been linked to cardiac disease in cats. One study of comparative concentrations of AGP in pleural effusions of different etiology in cats identified lower AGP concentrations in cardiogenic compared with infectious or neoplastic effusions, but no healthy controls were included.^29^ In our study, AGP appeared to be an independent risk predictor in CHF cats and it was significantly correlated with cTnI and NT‐proBNP. Thus, it may have potential as a prognostic cardiac biomarker. In humans, AGP is an independent prognosticator for cardiovascular mortality as well as all causes of mortality.^34^ The role of AGP as a prognostic marker in cats with heart disease should be further investigated.

The other 3 APPs evaluated in our study, including CRP, did not show significant differences in study cats. The most commonly studied APP in heart failure is CRP. In dogs, CRP has been shown to be increased in CHF and correlated with disease severity.^33^, ^36^, ^38^ It does not appear to be a major APP in cats,^64^ but interestingly, we identified significant positive correlations of CRP with renal markers. Our results suggest a link between the cardiorenal axis and an inflammatory response in cats with CHF, which merits further investigation.

One secondary finding of our study is that lower body weight was found to be an independent poor prognosticator in CHF cats. Weight loss with cardiac cachexia could be a logical explanation for the poorer survival. A previous study reported CHF cats with extreme body weights (either too low or too high) had poorer clinical outcomes.^51^ Therefore, balanced nutrition, aiming for a target weight within normal physiological range, is probably most beneficial for CHF cats in long‐term management.^66^

Another interesting finding was that cardiomyopathy phenotype did not affect the concentration of any investigated biomarker. Thus, these biomarkers potentially can serve as universal tools in studying different cardiomyopathies.

A multiple clinical variables‐based scoring approach has been advocated for risk stratification in humans with heart failure and preliminary evaluations look promising.^67^, ^68^ This approach could be a future direction in cats. The biomarkers we studied could contribute to such a scoring system. For example, the prognostic factors we identified (body weight, LA/Ao ratio, and serum AGP concentration) could be scored in individual patients. A cumulative quantitative score could be used as an objective risk assessment tool in CHF cats.

Our study had several limitations. It was a small study, which affected the power of statistical analysis, particularly the survival analysis. The longitudinal investigation included only a small number of animals. A larger scale, prospective, cohort study is necessary to corroborate our findings.

Although most data were prospective, in 9 cats the serum samples for the APP study were selected from service archives, and their NT‐proBNP measurements were acquired retrospectively.

Another limitation is that the effect of medications on biomarker concentrations cannot be assessed because all of the CHF cats had pretreatment. Medications such as loop diuretics can affect renal function, which can be a major contributor to cardiorenal biomarker increases in CHF cats. Nevertheless, effective treatment with diuretics in acute CHF can benefit renal function.^54^ Ideally, an untreated CHF population should be used to assess CvRD, which would help differentiate primary cardiac**‐**induced effects from treatment‐induced prerenal or renal azotemia. However, such a population is difficult to obtain in a referral clinic.

The APPs studied provide valuable information for cats with CHF, but the findings should be interpreted with caution. Considering the nature of APPs, species differences, occult or undetected comorbidities, biological variables and variability of test assays all might have influenced our findings.^24^, ^25^, ^61^ For example, dental disease in geriatric cats was a common incidental finding and undiagnosed dental disease could affect data interpretation. Occult or undiagnosed inflammatory conditions were challenging to eliminate without subjecting all candidate cats to exhaustive clinical diagnostic testing, which was not feasible. We do not recommend using APPs as a sole test in cats with CHF, but they may be helpful for understanding disease pathogenesis and could be useful for establishing disease prognosis in combination with other clinical assessments.

The exclusion criteria were based mainly on clinical records, which were not optimal, particularly for ruling out primary renal disease and inflammatory comorbidities. Ideally, complete urinalysis should have been performed to screen for CKD when the cats were first enrolled, despite the fact that there was no history of polyuria, polydipsia or abnormal kidneys based on palpation. However, excluding cardiac patients with renal disease might result in the exclusion of cats with CvRD. Another limitation of the exclusion process was that records of systolic BP were not available in 11 CHF and 3 preclinical cardiomyopathy cats. The reasons for the missing data include cats that would not tolerate BP measurement at presentation and in which no other clinical evidence indicated systemic hypertension; cats that had BP measured at the referring practice; and, cats that were screened but for which documentation was unavailable in the archives.

Full clinical records of the healthy control cats were not accessible because their serum samples were from a commercial source and echocardiography examination could not be performed in this group. Although their low serum NT‐proBNP concentrations (<30 pmol/L) suggested that occult heart disease was very unlikely, preclinical cardiomyopathy cannot be completely ruled out in these cats. The healthy controls also were significantly younger than the cardiomyopathy cats, and thus an age effect on biomarker concentration cannot be excluded. However, in the age and biomarker correlation assessment, except for a moderate age correlation observed for serum creatinine concentration and weak age correlations observed for LRG1 and SAA, the remaining biomarkers were not associated with age.

Inadequate serum sample volume was a problem in several cats, and thus not all cats could have all biomarkers measured. Nine serum samples for the APP tests were stored for 3 years and the effect of long‐term storage on biomarker concentration was not evaluated. The NT‐proBNP concentrations in our study were limited by the IDEXX Cardiopet assay detection range (ie, 24‐1500 pmol/L). Therefore, the median NT‐proBNP concentration in the CHF cats was underestimated. Lastly, in the APP component, a PCT assay designed for dogs was used, and species specificity could have influenced measured PCT results.

## 5 CONCLUSIONS

Our results support the presence of CvRD and systemic inflammation in cats with CHF. The findings suggest that SDMA, LRG1, SAA, and ceruloplasmin are promising novel biomarkers for CHF caused by primary cardiomyopathy in cats. A combination of clinical tests and serum biomarkers can be a future direction for risk stratification of disease. Our study provides new insights into biomarker research in cats, and the findings may benefit clinical management as well as fundamental research into CHF and cardiomyopathy in cats.

## CONFLICT OF INTEREST DECLARATION

The acute‐phase proteins were assayed at Life Diagnostic Inc.; the same company also produces the test assays.

## OFF‐LABEL ANTIMICROBIAL DECLARATION

Authors declare no off‐label use of antimicrobials.

## INSTITUTIONAL ANIMAL CARE AND USE COMMITTEE (IACUC) OR OTHER APPROVAL DECLARATION

The study obtained ethical approval from the University of Glasgow Veterinary Research Ethics Committee.

## HUMAN ETHICS APPROVAL DECLARATION

Authors declare human ethics approval was not needed for this study.

## Supporting information


**Appendix** **S1.** Supporting information tables
**Table S1.** Comparison of serum biomarker concentrations in CHF, preclinical cardiomyopathy and healthy control cats.
**Table S2.** Comparison of serum biomarker concentrations among different cardiomyopathy phenotypes.
**Table S3.** Univariate Cox proportional hazards analysis evaluating the effects of potential prognostic factors for CHF cats.
**Table S4.** Longitudinal data summary in CHF Cats‐NT‐proBNP.
**Table S5.** (1) Longitudinal data summary in survived CHF Cats‐SDMA and creatinine. (2) Longitudinal data summary in non‐survived CHF Cats‐SDMA and creatinine.
**Table S6.** Longitudinal data summary in CHF Cats‐APPs.
**Table S7.** Classification and diagnostic criteria for feline cardiomyopathy.
**Table S8.** Left ventricle internal diameters of CHF and preclinical cardiomyopathy cats.
