# Supplementary material for: Cardiovascular‐renal axis disorder and acute‐phase proteins in cats with congestive heart failure caused by primary cardiomyopathy
Source: J Vet Intern Med. 2020 May 12;34(3):1078–90. doi: 10.1111/jvim.15757 (PMC7255652; doi:10.1111/jvim.15757)
Supplement: Supplementary file 1 — Appendix S1. Supporting information tables Table S1. Comparison of serum biomarker concentrations in CHF, preclinical cardiomyopathy and healthy control cats. Table S2. Comparison of serum biomarker concentrations among different cardiomyopathy phenotypes. Table S3. Univariate Cox proportional hazards analysis evaluating the effects of potential prognostic factors for CHF cats. Table S4. Longitudinal data summary in CHF Cats‐NT‐proBNP. Table S5. (1) Longitudinal data summary in survived CHF Cats‐SDMA and creatinine. (2) Longitudinal data summary in non‐survived CHF Cats‐SDMA and creatinine. Table S6. Longitudinal data summary in CHF Cats‐APPs. Table S7. Classification and diagnostic criteria for feline cardiomyopathy. Table S8. Left ventricle internal diameters of CHF and preclinical cardiomyopathy cats. [file JVIM-34-1078-s001.pdf]

Supplementary Table 1. Comparison of serum biomarker concentrations in CHF, preclinical cardiomyopathy and healthy control cats

	CHF		Preclinical		Healthy Control		
**Biomarker**	**n**	**Median (IQR)**	**n**	**Median (IQR)**	**n**	**Median (IQR)**	**P value***
NT-ProBNP (pmol/l)	19	1293[a] (780, >1500)	11	282[b] (76, 532)	20	<24[c] (<24, <24)	< .001
SDMA(ug/dl)	14	18.5[a] (12.0, 23.0)	5	11.0[b] (8.5, 13.5)	19	9.0[b] (8.0, 12.0)	< .001
Creatinine(µmol/l)	14	159[a] (117, 195)	5	127[a, b] (115, 181)	19	95[b] (80, 120)	<.001
AGP (µg/ml)	15	302 (240, 626)	9	187 (147, 325)	16	290 (187, 454)	.165
CRP (µg/ml)	15	360 (226, 509)	9	290 (187, 454)	16	316 (218, 388)	.192
Hp (µg/ml)	15	239 (32, 321)	9	141 (117, 217)	16	163 (57, 231)	.328
LRG1 (µg/ml)	15	8.9[a] (6.4, 10.8)	9	5.3[a, b] (4.3, 7.0)	16	3.3[b] (2.7, 4.4)	.020
SAA (µg/ml)	15	1.34[a] (1.00, 11.27)	9	0.79[b] (0.56, 1.00)	16	0.75[b] (0.64, 1.07)	.003
PCT (ng/ml)	15	0 (0, 50.6)	9	0 (0, 5.7)	16	0 (0, 20.0)	.717
Ceruloplasmin (µg/ml)	15	326[a] (272, 419)	9	227[b] (194, 268)	16	283[b] (241, 312)	< .001
cTnI (ng/ml)	5	1.11[a] (1.03, 1.69)	4	0.37[b] (0.12, 0.64)	0		.010

* Medians without superscripts in common were significantly different (P < .05) based on pairwise t tests with Bonferroni correction of P values.

Supplementary Table 2. Comparison of serum biomarker concentrations among different cardiomyopathy phenotypes

	HCM (n = 22)		UCM (n = 8)		Other* (n = 7)		
**Biomarker**	**n**	**Median (IQR)**	**n**	**Median (IQR)**	**n**	**Median (IQR)**	**P value†**
NT-ProBNP (pmol/l)	20	536 (214, 1426)	8	1397 (900, >1500)	6	1089 (721, >1500)	.054
SDMA (ug/dl)	11	14.0 (11.0, 23.0)	7	20.0 (13.0, 21.0)	5	13.0 (11.0, 19.5)	.599
Creatinine (µmol/l)	11	154 (104, 185)	7	194 (161, 201)	5	116 (98, 151)	.050
AGP (µg/ml)	18	252 (165, 629)	7	297 (239, 520)	3	407 (302, 416)	.840
CRP (µg/ml)	18	325 (234, 402)	7	394 (360, 711)	3	476 (193, 490)	.577
Hp (µg/ml)	18	187 (104, 332)	7	292 (32, 472)	3	286 (272, 492)	.544
LRG1 (µg/ml)	18	7.0 (5.0, 10.3)	7	8.9 (6.4, 10.8)	3	8.4 (6.4, 10.8)	.768
SAA (µg/ml)	18	1.0 (0.79, 3.21)	7	1.10 (0.73, 11.83)	3	1.48 (1.12, 1.70)	.695
PCT (ng/ml)	18	0 (0, 11.6)	7	0 (0, 12.4)	3	0 (0, 96.0)	.980
Ceruloplasmin (µg/ml)	18	272 (226, 391)	7	326 (263, 481)	3	468 (294, 652)	.129

*Combined group of DCM (n = 1) and RCM (n = 6).

Supplementary Table 3. Univariate Cox proportional hazards analysis evaluating the effects of potential prognostic factors for CHF cats

Variable/level	n	PE (β̂)	HR (95% CI)	P value (Wald)
Age				.501
<5 years	8	0.751	2.12 (0.51, 8.90)	.305
5-10 years	7	0.825	2.28 (0.50, 10.4)	.287
>10 years	10	Referent		
Sex				
Male	17	-0.138	0.87 (0.28, 2.76)	.815
Female	8	Referent		
Weight				
<4.5 kg	11	0.431	1.54 (0.49, 4.86)	.463
≥4.5 kg	13	Referent		
LA/Ao ratio	25	1.131	3.10 (1.36, 7.05)	.007**
Unstable CHF	25	2.486	12.0 (1.53, 95.5)	.018*
Arrhythmia by auscultation	18	2.644	14.1 (1.68, 118)	.015*
Gallop sounds	24	1.631	5.11 (1.18, 22.1)	.029*
AGP	19	0.005	1.005 (1.001, 1.0029)	.007**

Results are presented for signalment and only those other variables with $P < .05$. ** indicates statistical significance at $P < .01$; * indicates statistical significance at $P < .05$.

Supplementary Table 4. Longitudinal data summary in CHF Cats-NT-proBNP

CHF Survivor (n = 6)

**Cat ID**	Day 0	Day 1-14	Day 15-30	Day 31-90	Day 91-180	Day 181-365	Overall Trend
**No.1**	>1500					>1500	Persistently high
**No.2**	578					633;<br>530	Stable
**No.4**	213				45		↓
**No.7**	882		982				Stable
**No.10**	1477	578		1282			↓↑
**No.12**	>1500			950			Stable or ↓

CHF Non-Survivor (n = 6)

**Cat ID**	Day 0	Day 1-14	Day 15-30	Day 31-90	Day 91-180	Day 181-365	Overall Trend
**No.3**	>1500			950			Stable or ↓
**No.5**	>1500	692					Stable or ↓
**No.6**	>1500		>1500	>1500;<br>>1500	>1500		Persistently high
**No.8**	>1500		>1500				Persistently high
**No.9**	>1500	>1500					Persistently high
**No.11**	>1500		>1500				Persistently high

Serum biomarker changing trends post initial diagnosis were illustrated. Serum biomarker changing trends post initial diagnosis are illustrated. Day 0 was defined as the 1st day of blood sampling. For the overall trend summary, a NT-proBNP value >1500 pmol/l is described as 'high'; a more than 60% positive or negative change from the previous measurement is described as 'increased' or 'decreased'; less than 60% of concentration change from the previous measurement is described as 'stable'; ↓, decreased; ↑, increased; ↓↑, firstly decreased then followed by an increase; ↑↓, firstly increased then followed by a decrease.

Supplementary Table 5-1. Longitudinal data summary in survived CHF Cats-SDMA and creatinine

CHF Survivor (n = 6)																
**Cat ID**	**Day 0**		**Day 1-14**		**Day15-30**		**Day31-90**		**Day91-180**		**Day 181-365**		**Initial Assessment**		**Overall Trend**	
	**SDMA**	**Cr**	**SDMA**	**Cr**	**SDMA**	**Cr**	**SDMA**	**Cr**	**SDMA**	**Cr**	**SDMA**	**Cr**	**SDMA**	**Cr**	**SDMA**	**Cr**
No.1	12	154									*15	*135	Normal	Normal	↑ to high	↓
No.2	13	116									12; *17	90; *139	Normal	Normal	↑ to high	↓↑
No.4	/	/							11	128			/	/	/	/
No.7	17	194			19	208							High	High	↑	↑
No.10	10	157	8	122			9	126					Normal	Normal	↓↑	↓↑
No.12	16	183					10	148					High	High	↓ to normal	↓ to norma

Unit: SDMA-ug/dl; creatinine-umol/l. * SDMA and creatinine conflicting data pairs. Cat No.4 Day 0 data missing was due to a technical reason. SDMA >14 ug/dl or creatinine >165 umol/l was defined as 'high'. ID, identification; ↓, decreased; ↑, increased; ↓↑, firstly decreased then followed by an increase; ↑↓, firstly increased then followed by a decrease.

Supplementary Table 5-2. Longitudinal data summary in non-survived CHF Cats-SDMA and creatinine

CHF Non-Survivor (n = 6)

**Cat ID**	**Day 0**		**Day 1-14**		**Day15-30**		**Day31-90**		**Day91-180**		**Day 181-365**		**Initial Assessment**		**Overall Trend**	
	**SDMA**	**Cr**	**SDMA**	**Cr**	**SDMA**	**Cr**	**SDMA**	**Cr**	**SDMA**	**Cr**	**SDMA**	**Cr**	**SDMA**	**Cr**	**SDMA**	**Cr**
No.3	*20	*136									*21; 31	*165; 211	High	Normal	↑	↑ to high
No.5	*14	*185	10	125									Normal	High	↓	↓ to norma
No.6	21	274			22	223	22; 22	235; 189	*36	*116			High	High	↑	↓ to norma
No.8	*19	*65			*22	*72							High	Normal	↑	↑
No.9	13	161	12	155									Normal	Normal	↓	↓
No.11	*25	*104			14	75							High	Normal	↓ to normal	↓

Unit: SDMA-ug/dl; creatinine-umol/l. * SDMA and creatinine conflicting data pairs. Cat No.4 Day 0 data missing was due to a technical reason. SDMA >14 ug/dl or creatinine >165 umol/l was defined as 'high'. ID, identification; ↓, decreased; ↑, increased; ↓↑, firstly decreased then followed by an increase; ↑↓, firstly increased then followed by a decrease.

Supplementary Table 6. Longitudinal data summary in CHF Cats-APPs

**CHF Survivor (n=6)**							
**Cat ID**	**AGP**	**CRP**	**Hp**	**LRG1**	**SAA**	**PCT**	**Ceruloplasmin**
**No.1**	↓	↓	↑	↓	↓	_	↓
**No.2**	↓↑	↓	↓	↓	↓	_	↓↑
**No.4**	/	/	/	/	/	/	/
**No.7**	/	/	/	/	/	/	/
**No.10**	↑	↓↑	↑↓	↑	↑	↑	↑
**No.12**	↓	↓	↓	↓	↑	_	↓
**CHF Non-Survivor (n=6)**							
**Cat ID**	**AGP**	**CRP**	**Hp**	**LRG1**	**SAA**	**PCT**	**Ceruloplasmin**
**No.3**	↑	↓	↓	↑	↑	_	↓
**No.5**	↓	↓	↓	↓	↓	_	↓
**No.6**	↑	↑	↓	↑	↑	↓	↑
**No.8**	↑	↓	↓	↓	↓	_	↓
**No.9**	↓	↓	_	↓	↑	_	↓
**No.11**	/	/	/	/	/	/	/

Day 0 was defined as the 1st day of blood sampling. Trends of change are summarized for each APP in each individual cat. Note each cat demonstrated diverse changing patterns among APP expression; except in Cat No. 5, all the APPs decreased in subsequent measurements compared with the measurements at the initial time point. Cats No. 4, No. 7 and No. 11 could not have serial APPs measured due to serum sample volume limitations; cats No. 7 and No. 11 had the Day 0 concentration measured but not at subsequent time points. In the APPs longitudinal study, there were 1-2 follow up time points (not including Day 0). ↓, decreased; ↑, increased; ↓↑, firstly decreased then followed by an increase; ↑↓, firstly increased then followed by a decrease.

Supplementary Table 7. Classification and diagnostic criteria for feline cardiomyopathy

CM Classification	Diagnostic Criteria Based on Echocardiography
HCM	Left ventricular hypertrophy was defined as an end-diastolic maximal thickness of the left ventricular free wall (LVFWd) or interventricular septum (IVSd) of ≥ 6 mm; and/or evidence of focal hypertrophy in the left ventricle. HOCM was characterized by presence of left ventricle out flow tract obstruction [39,42].
RCM	Characterized by marked bi-atrial enlargement without significant myocardial hypertrophy, normal or mildly reduced myocardial systolic function, and diastolic dysfunction i.e. a restrictive left ventricular filling pattern confirmed with Doppler echocardiography [40].
DCM	Characterized by dilation of the left ventricular lumen and decreased myocardial function. In the current study the following cut-offs were used: left ventricular internal diameter end systole (LVIDs) > 14 mm, LV FS < 28% [44].
ARVC	Marked right side cardiac chamber enlargement particularly right ventricular dilation with thinning of the myocardium, myocardium replacement by fibrotic/fatty tissue, right atrial dilation and an apical ventricular aneurism may also be present [43].
UCM	Myocardial abnormalities that do not readily fit into any of the classifications defined above [41].

Supplementary Table 8. Left ventricle internal diameters of CHF and preclinical cardiomyopathy cats

	CHF (n = 25)		Preclinical (n = 12)	
Variable	n/d	Median (IQR)	n/d	Median (IQR)
LVIDd (mm)	23/25	14.0 (11.5, 18.2)	9/12	13.7 (11.3, 14.5)
LVIDs (mm)	23/25	8.6 (6.4, 13.7)	9/12	6.9 (5.9, 7.9)

CHF, congestive heart failure; n/d, numerator/denominator; IQR, interquartile range; LVIDd, left ventricular internal diameter end diastole; LVIDs, left ventricular internal diameter end systole.
